# Prize Essays: American Medical Editors’ Association

**Published:** 1872-07

**Authors:** 


Prize Essays.—The Committee of the American Medical Editors' Association, appointed on a proper subject for prize essays, have recommended the following for the prize to be awarded in May, 1873:—

"The Pathology and Treatment of Diseases of the Ovaries."

And the prize to be awarded in May, 1874, the following:—

"At what stages of Pulmonary Tuberculosis is a change of climate desirable; what are the principles which should govern us in choosing the kind of change to be made; and the best localities in North America to send patients of this class ?"

The prize offered is $100, and competition is open to all medical editors. *Boston M. & S. Journal.*

